# Odorant Inhibition in Mosquito Olfaction

**DOI:** 10.1016/j.isci.2019.07.008

**Published:** 2019-07-12

**Authors:** Pingxi Xu, Young-Moo Choo, Zhou Chen, Fangfang Zeng, Kaiming Tan, Tsung-Yu Chen, Anthony J. Cornel, Nannan Liu, Walter S. Leal

**Affiliations:** 1 Department of Molecular and Cellular Biology, University of California-Davis, Davis, CA 95616, USA; 2 Department of Entomology and Plant Pathology, Auburn University, Auburn, AL 36489, USA; 3 Center for Neuroscience, Department of Neurology, University of California-Davis, Davis, CA 95616, USA; 4 Department of Entomology and Nematology, University of California-Davis, Davis, CA 95616, USA

**Keywords:** Biological Sciences, Neuroscience, Sensory Neuroscience

## Abstract

How chemical signals are integrated at the peripheral sensory system of insects is still an enigma. Here we show that when coexpressed with Orco in *Xenopus* oocytes, an odorant receptor from the southern house mosquito, CquiOR32, generated inward (regular) currents when challenged with cyclohexanone and methyl salicylate, whereas eucalyptol and fenchone elicited inhibitory (upward) currents. Responses of CquiOR32-CquiOrco-expressing oocytes to odorants were reduced in a dose-dependent fashion by coapplication of inhibitors. This intrareceptor inhibition was also manifested *in vivo* in fruit flies expressing the mosquito receptor CquiOR32, as well in neurons on the antennae of the southern house mosquito. Likewise, an orthologue from the yellow fever mosquito, AaegOR71, showed intrareceptor inhibition in the *Xenopus* oocyte recording system and corresponding inhibition in antennal neurons. Inhibition was also manifested in mosquito behavior. Blood-seeking females were repelled by methyl salicylate, but repellence was significantly reduced when methyl salicylate was coapplied with eucalyptol.

## Highlights

• We found dual inhibitory/excitatory odorant receptors (ORs) in mosquitoes
• Inhibitory and endogenous ORs coexpressed in flies showed lateral inhibition
• The bipolar nature of these inhibitory ORs was displayed in electrophysiology
• The duality excitation/inhibition was also manifested in mosquito behavior

## Graphical Abstract

**Figure:**
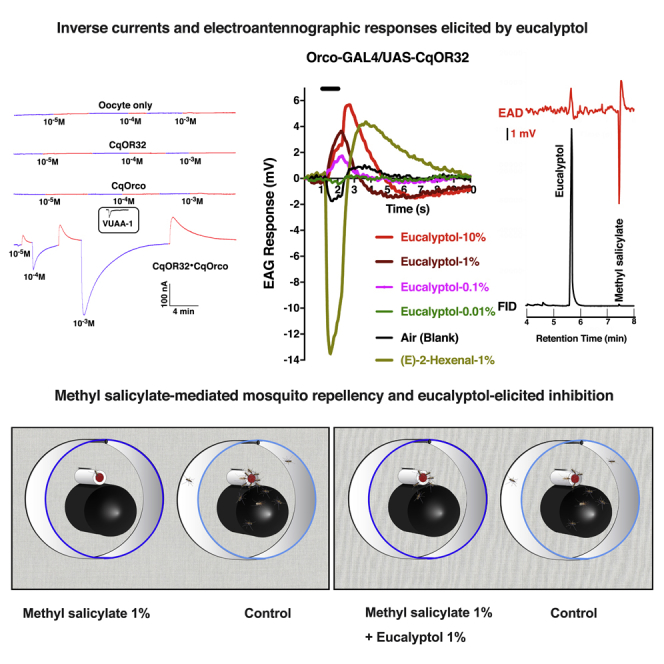

## Introduction

Integration of chemical signals at the peripheral sensory system (antennae, maxillary palps, and proboscis) remains one of the least understood mechanisms of insect olfaction, particularly in mosquitoes. Despite the great progress made in the last 2 decades in understanding how receptors form the basis of chemosensory perception in insects, how olfactory (as well as taste) signals integrate at the periphery remains an enigma (Joseph and Carlson, 2015). “It is as if a new continent has been discovered but only the coastline has been mapped” (Joseph and Carlson, 2015). In the largest majority of reported cases (Hill et al., 2009, Syed and Leal, 2009, Liu et al., 2013, Ye et al., 2016), antennal neurons of *Cx. quinquefasciatus* displayed excitatory responses (increased spike frequency upon stimulus), but evidence for inhibitory responses (reduction in spontaneous activity upon stimulus), already known for *Ae. aegypti* (Ghaninia et al., 2007), is now emerging for *Cx. quinquefasciatus* (Ye et al., 2016). It has been observed in moths (Kaissling, 1996), beetles (Nikonov and Leal, 2002), the fruit (vineger) fly (Su et al., 2012), and mosquitoes (Tauxe et al., 2013) that activation (firing) of one neuron interferes with signaling of other olfactory receptor neurons (ORNs; also referred to as olfactory sensory neurons). It has also been reported that a single compound (iodobenzene) caused reduction of nerve impulse (inhibition) followed by a transient post-stimulus excitation (de Brito sanchez and Kaissling, 2005). Although Carlson and collaborators elegantly demonstrated that in the fruit fly lateral inhibition is most likely mediated by ephaptic coupling (Su et al., 2012), the complete ensemble of the molecular mechanism(s) of inhibition at the peripheral olfactory system of mosquitoes remains *terra incognita*. A simple explanation of the ephaptic coupling is that, upon (continuous) stimulation of an ORN, the (external) potential (of the sensillum lymph surrounding dendrites) declines. Consequently, per channel current generated by a cocompartmentalized neuron (when stimulated by its cognate ligand) is reduced (Van Naters, 2013). This scenario argues that the firing of a neuron causes reduced spike frequency by a colocated neuron because of the close apposition (ephaptic, Greek for “to touch”) of their neuronal processes. Although ephaptic coupling could explain lateral inhibition, other mechanisms of intraneuron inhibition may exist. While de-orphanizing odorant receptors (ORs) expressed predominantly in *Cx. quinquefasciatus* female antennae, we serendipitously recorded currents from an OR that generate inhibition in response to certain odorants. Further studies unraveled a hitherto unknown mechanism of peripheral, intrareceptor inhibition in mosquito olfaction.

## Results and Discussion

### Recordings of Inward Currents and Currents in Upward Direction

**Figure 1:**
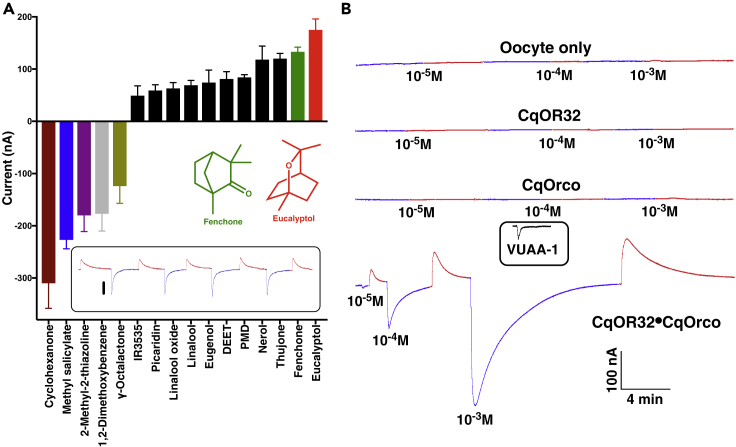
Recordings and Quantification of Inward and Inhibitory Currents (A) CquiOR32-CquiOrco-expressing oocytes responses to ligands at 1 mM. Error bars represent SEM (n = 3-4). For clarity, bars representing reverse and inward currents are displayed upward and downward, respectively. The following compounds are not displayed for conciseness. Inward current-eliciting compounds: γ-hexalactone, 115 ± 14 nA (all data in absolute values); guaiacol, 101 ± 11 nA; acetophenone, 58 ± 12 nA; 2-butanone, 45 ± 7 nA; 2-phenylethanol, 44 ± 9 nA. Inhibitory compounds: α-terpinene, 43 ± 5 nA; terpinolene, 38 ± 8 nA (for a complete list, see Table S1). *Inset*: Continuous recordings from a single oocyte expressing CquiOR32-CquiOrco and repeatedly stimulated with 1 mM eucalyptol (reverse peaks; traces in maraschino) and 0.1 mM methyl salicylate (inward peaks; traces in blueberry). (B) Control experiments with oocytes only and oocytes expressing only CquiOR32, CquiOrco, or a combination of CquiOR32 and CquiOrco. Response of a CquiOrco-expressing oocyte to Orco agonist, VUAA-1, is displayed in an inset. For clarity, the traces before, during, and after stimulus are displayed with the same color as the bars in (A). Specifically, methyl salicylate in blueberry (CYMK, Cyan 87%, Magenta 61%, Yellow 0%, Black 0%) and eucalyptol in maraschino (CYMK, Cyan 0%, Magenta 81%, Yellow 94%, Black 1%). Stimuli were delivered for 2 s.

In our attempts to de-orphanize ORs from the southern house mosquito, *Cx. quinquefasciatus*, we challenged *Xenopus* oocytes coexpressing CquiOR32 along with the obligatory coreceptor Orco with a panel of more than 200 compounds, including mostly physiologically and behaviorally relevant compounds (Xu et al., 2014). Because CquiOR32 is predominantly expressed in female antennae (Figure S1), we reasoned that this receptor might be involved in the reception of attractants or repellents. CquiOR32-CquiOrco-expressing oocytes generated dose-dependent inward (regular) currents when challenged with various odorants, including cyclohexanone, methyl salicylate, and 2-methyl-2-thiazoline (Figure 1A, Table S1). Interestingly, however, eucalyptol, fenchone, DEET, picaridin, IR3535, PMD, and other compounds generated currents in reverse direction (Figure 1A and Table S1) thus resembling inverse agonists. These unusual currents of reverse direction were reproducible, and no indication was found of adaptation (Figure 1A, inset). No currents were recorded when oocytes alone or oocytes expressing only CquiOR32 or only CquiOrco were challenged either with methyl salicylate or eucalyptol (Figure 1B). However, CquiOR32-CquiOrco-expressing oocytes responded to both compounds. Methyl salicylate elicited inward currents, and eucalyptol generated currents in the reverse direction (Figure 1B) in a dose-dependent manner (Figures S2 and S3, respectively).

### Current-Voltage Curves Suggest that OR32-Orco Forms Nonselective Cation Channels

**Figure 2:**
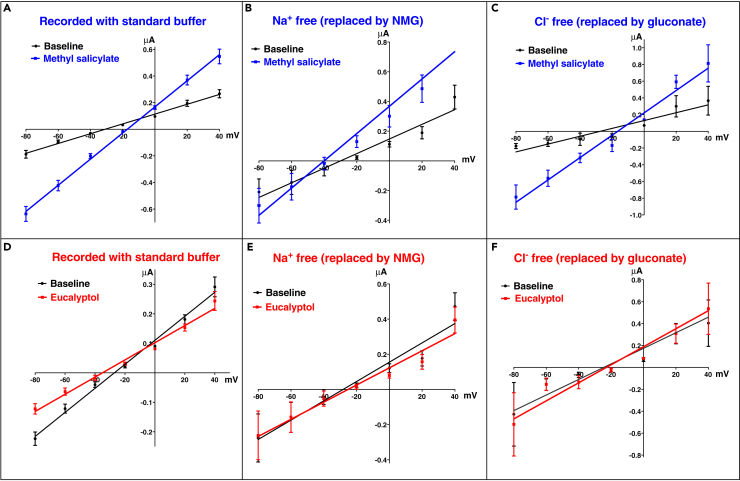
Current-Voltage (I-V) Curves for CquiOR32-CquiOrco Expressed in *Xenopus* Oocytes Recordings with methyl salicylate (blueberry) and eucalyptol (maraschino) at 1 mM, with voltage clamped at −80, −60, −40, −20, 0, +20, and +40 mV and using regular and modified buffers. (A and D, n = 9) standard buffer; (B and E, n = 5) Na^+^ free buffer; (C and F, n = 3) Cl^−^ free buffer. Error bars represent SEM.

To gain better insights into possible mechanism(s) of these currents in reverse direction, we obtained current-voltage (I-V) curves from oocytes expressing wild-type (WT) CquiOR32-CquiOrco with voltage clamped at −80, −60, −40, −20, 0, +20, and +40 mV and using methyl salicylate and eucalyptol as stimuli (Figures 2A and 2D, respectively). Similar recordings were performed using N-methyl-d-glucamine chloride (NMG) as a source of bulky, impermeable mono cation (Figures 2B and 2E) or with sodium gluconate buffer (a source of bulky, impermeable anions) instead of NaCl (Figures 2C and 2F). These data corroborate that OR32-Orco forms nonselective cation channels (Wicher et al., 2008, Smart et al., 2008, Sato et al., 2008), as indicated by the reversal potential shift to more negative voltage upon replacement of Na^+^ by less permeable NMG (Figures 2A and 2B). An examination of the first and third quadrants for each graphic in Figure 2 shows that I-V curves elicited by methyl salicylate (Figures 2A–2C) are above baseline and below control I-V curves for positive and negative voltages, respectively (a common pattern of inward currents), consistent with a potentiation of the control current by methyl salicylate. By contrast, I-V curves generated with eucalyptol, albeit not robust, are below and above control I-V curves for positive and negative holding potentials, respectively (Figures 2D and 2E), suggesting an inhibition effect. In the presence of eucalyptol, replacing Na^+^ with NMG had a minor effect on the I-V curves (Figure 2E), whereas replacing Cl^−^ with gluconate seems to suggest that Cl^−^ is implicated in this inhibition (Figure 2F).

### Eucalyptol-Induced Inhibition in Oocytes

We then surmised that these “inhibitors” might modulate responses to odorants when these two types of stimuli are simultaneously delivered to receptors. First, we challenged a CquiOR32-CquiOrco-expressing oocyte with methyl salicylate (0.1 mM) and then eucalyptol (1 mM) and recorded regular and reverse currents, respectively (Figure S4). Then we challenged the same oocyte preparation with mixtures of methyl salicylate and eucalyptol. Binary mixtures with higher doses of eucalyptol elicited reverse currents. Inhibition was also observed with lower doses of eucalyptol, which generated dose-dependent reduced inward currents. A continuous trace displayed in Figure S4 shows no difference in the responses to methyl salicylate before and after these tests thus ruling out adaptation. Taking together, these findings suggest that “intrareceptor” inhibition occurred consistently with outward currents previously recorded from antennal neurons of the vinegar fly, *Drosophila melanogaster* (Cao et al., 2017).

### Inhibition in the Antennae of Flies Expressing CquiOR32

**Figure 3:**
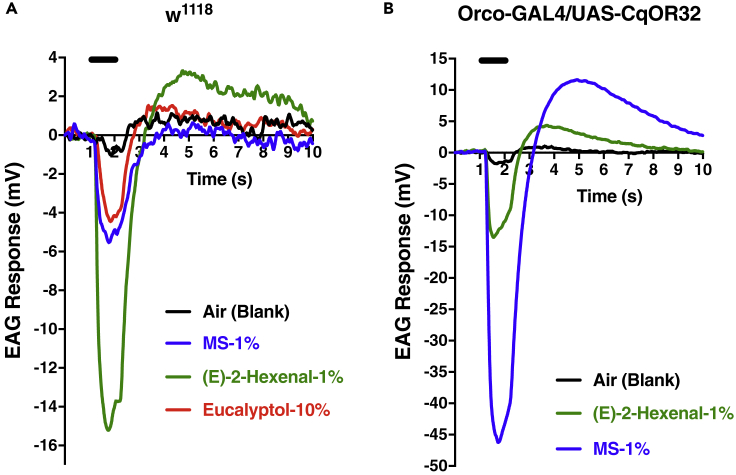
Electroantennogram (EAG) Obtained with Wild-Type and Transgenic Flies Responses elicited by 1% methyl salicylate (MS) and 1% (*E*)-2-hexenal in the antennae of w^1118^ (A) and Orco-GAL4/UAS-CqOR32 flies. Note the unusual robust response elicited by MS in transgenic flies (B) due to overexpression of CquiOR32.

**Figure 4:**
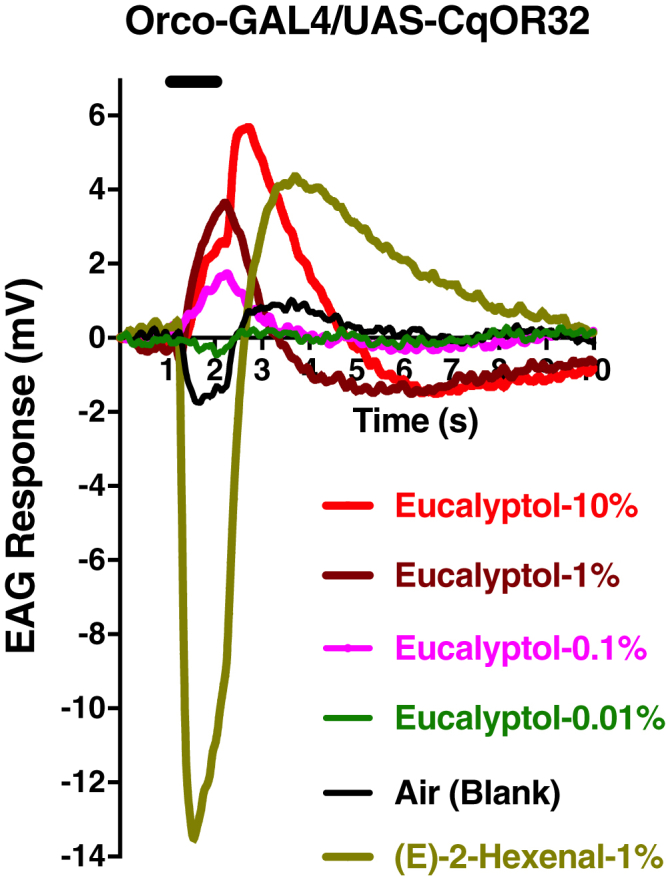
EAG Obtained by Challenging Orco-GAL4-CquiOR32 Flies with Eucalyptol While blank generate a normal downward deflection, eucalyptol elicited reverse responses in a dose-dependent manner.

**Figure 5:**
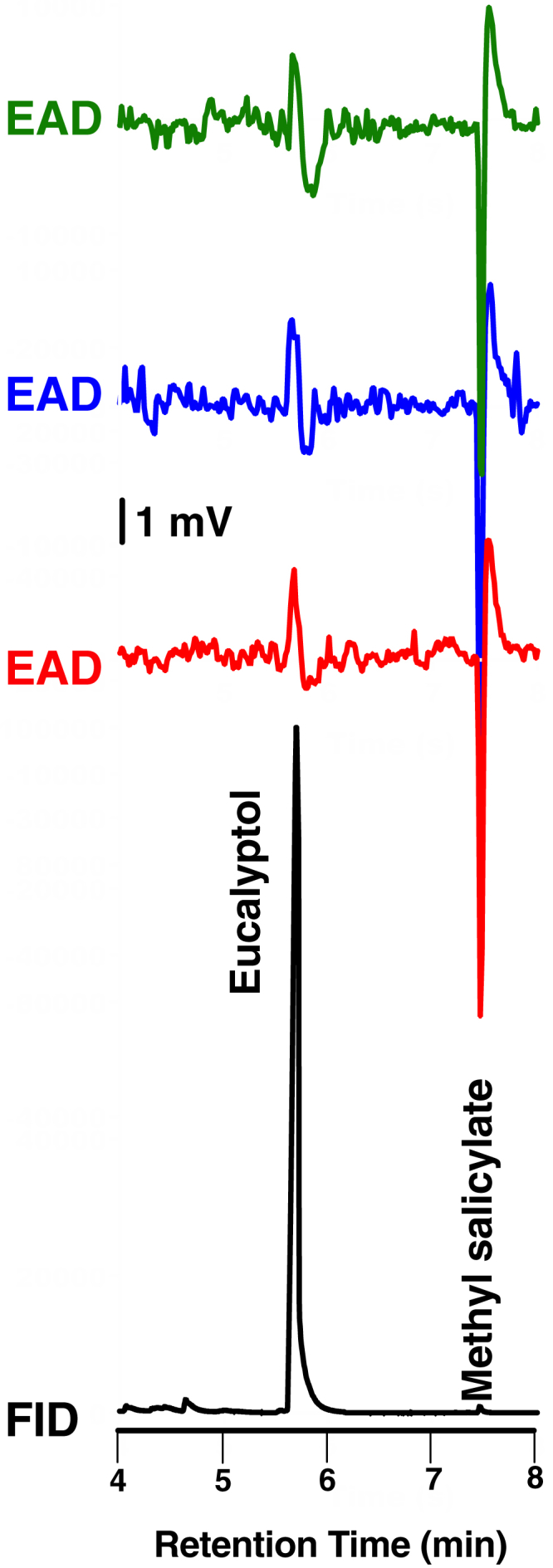
Antennal Response Obtained with Gas Chromatography (GC) Linked to Electroantennographic (EAD) Detection Response of the flame ionization detector (FID) was synchronized with EAD responses. For clarity only one trace of FID response is provided in these three replicates. Each EAD replicate is displayed in a different color. Methyl salicylate, 10 ng; eucalyptol 0.5 μg.

To test whether the inhibitory responses were manifested *in vivo* at the periphery of the olfactory system, we generated transgenic flies, with CquiOR32 expression driven by DmelOrco promoter, and recorded electroantennogram (EAG) responses by using a standard method (Ueira-Vieira et al., 2014). Not surprisingly, control flies (w^1118^) gave strong response to (*E*)-2-hexenal and weak response to methyl salicylate (Figure 3A), whereas Orco-Gal4/UAS-CqOR32 flies gave robust response to methyl salicylate, with the strong response to (*E*)-2-hexenal unchanged (Figure 3B). w1118 flies gave very weak responses to eucalyptol at high doses, but interestingly Orco-Gal4/UAS-CqOR32 flies generated dose-dependent, inverse EAG responses (Figure 4). To further scrutinize this unusual reverse EAG responses, we used gas chromatography (GC) with electroantennographic detection (EAD). In GC-EAD analyses, injected mixtures (e.g., eucalyptol and methyl salicylate) are separated by GC and subjected to antennal preparations under the same condition thus ruling out any possibility of mechanical interference and minor sample contamination. Here, methyl salicylate responded with regular EAG responses, i.e., with the first phase (downward), which is referred to as rise of the receptor potential, and the second phase starting at the end of the stimulus, commonly referred to as the decline of the receptor potential (upward, return to the baseline). This is analogous to the depolarization, repolarization, and hyperpolarization of a nervous impulse. As opposed to methyl salicylate, eucalyptol consistently gave inverse EAD responses (upward then downward) (Figure 5) thus corroborating what we observed in EAG analyses (see earlier discussion).

**Figure 6:**
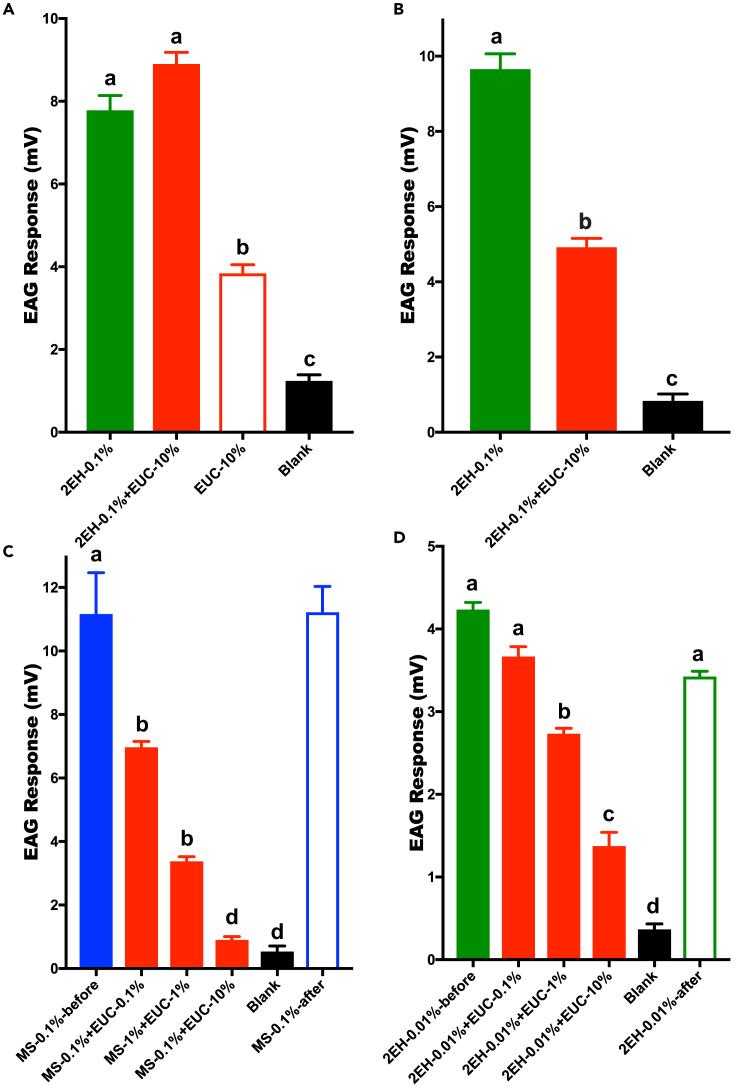
Quantification of EAG Responses Effect of eucalyptol (EUC) on the responses of (A) w^1118^ and (B–D) Orco-GAL4/UAS-CqOR32 flies to (E)-2-hexenal (2EH) and methyl salicylate (MS). Error bars represent SEM.

Next, we recorded EAG responses when flies were challenged with odorants and an inhibitor. First, we compared the response of w^1118^ and Orco-Gal4/UAS-CqOR32 flies to (*E*)-2-hexenal when it was delivered alone or in combination with eucalyptol. EAG responses from w^1118^ flies to 0.1% (*E*)-2-hexenal alone or in combination with 10% eucalyptol did not differ significantly (Figure 6A). By contrast, EAG responses from Orco-Gal4/UAS-CqOR32 flies to 0.1% (*E*)-2-hexenal plus 10% eucalyptol were significantly lower than those elicited by 0.1% (*E*)-2-hexenal alone (Figure 6B). We then examined the dose-dependent effect of this inhibition by using Orco-Gal4/UAS-CqOR32 flies. Robust responses to 0.1% methyl salicylate were reduced in a dose-dependent manner with the addition of eucalyptol (Figure 6C) but remained unchanged at the end of the tests. Likewise, EAG responses to 0.01% (*E*)-2-hexenal were reduced when coapplied with eucalyptol (0.1, 1, and 10%) (Figure 6D). Of note, (*E*)-2-hexenal does not activate CquiOR32 (Table S1, entry #136). Such inhibition presumably results from CquiOR32 indirectly inhibiting responses of the fly endogenous receptors (e.g., DmelOR7a) to (*E*)-2-hexenal. In these continuous experiments, a small difference between EAG responses before and after costimulus tests may be due to loss of this volatile semiochemical from the cartridge rather than adaptation. Similar inhibition was observed when 2-heptanone was applied alone or coapplied with eucalyptol (Figure S5). Taken together, these results further suggest that intrareceptor inhibition occurs *in vivo* as indicated by the inhibitory effect of eucalyptol on methyl salicylate responses. Additionally, the effect of eucalyptol on the response to (*E*)-2-hexenal suggests that intraneuronal inhibition occurred. A few lines of evidence support this hypothesis. First and foremost, eucalyptol does not cause inhibition in control flies (Figure 6A) and (*E*)-2-hexenal does not activate CquiOR32 (Table S1). The simplest explanation is that, in Orco-Gal4/UAS-CqOR32 flies, all endogenous receptors are coexpressed with CquiOR32. Thus, CquiOR32 response to eucalyptol interferes with the response of DmelOR7a (in ab4A neuron) to (*E*)-2-hexenal. In short, inhibitor and agonist are likely to be acting on different receptors in the same neurons, thus an intraneuron inhibition. To further test the notion of intraneuronal inhibition, we turned to single sensillum recordings (SSRs).

### Inhibition Is Also Manifested in Single Sensillum Recordings

**Figure 7:**
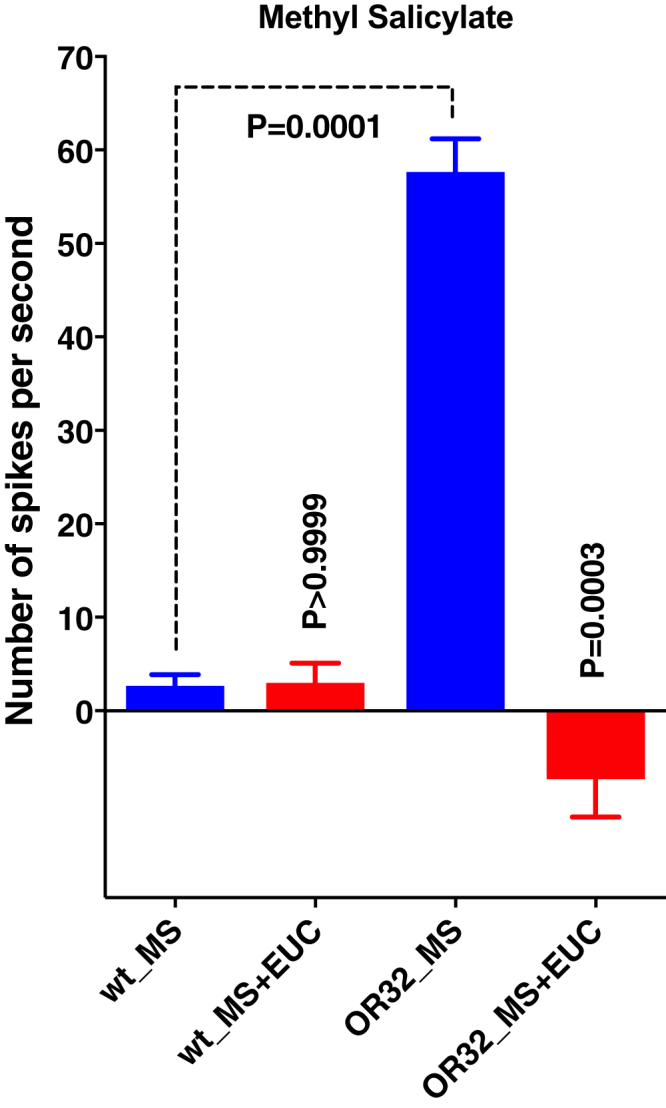
Quantification of Single Sensillum Recordings from ab4 sensilla in Wild-Type and Transgenic Flies Basal response to methyl salicylate (MS) recorded from WT flies did not differ when this odorant (0.1%) was delivered alone or codelivered with eucalyptol (EUC, 1%) (p > 0.9999). Spike frequency recorded from CquiOR32 flies were significantly higher than those obtained with WT flies (p = 0.0001). When codelivered with EUC (1%), the spike frequency was significantly reduced (p = 0.0003) to a level below the spontaneous activity. Error bars represent SEM.

**Figure 8:**
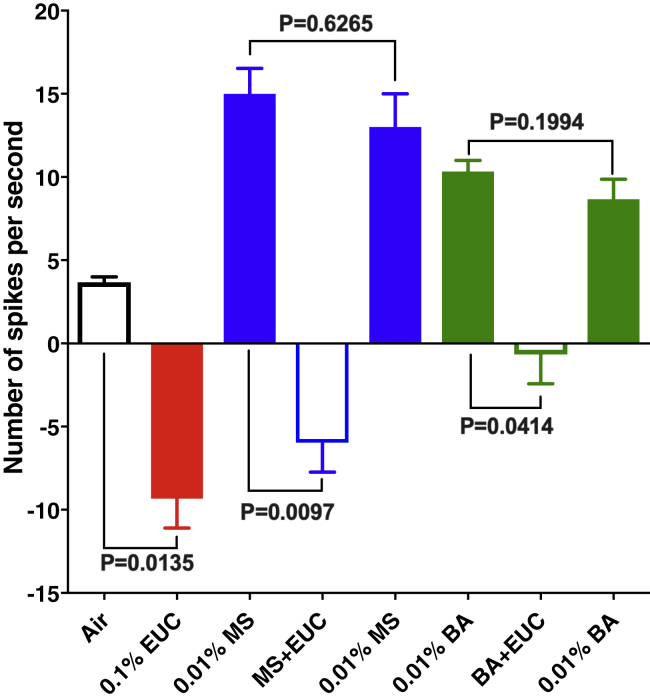
Quantification of Single Sensillum Recordings from ab7 Sensilla in Orco-GAL4/UAS-CqOR32 Flies Responses to 0.1% eucalyptol (EUC), 0.01% methyl salicylate (MS) before and after stimulus with a mixture of 0.01% MS plus 0.1% EUC, and 0.01% butyl acetate (BA) before and after stimulus with 0.01% BA plus 0.1% EUC. Error bars represent SEM.

The best ligand for ab4A, the neuron in ab4 sensilla with a large spike amplitude, is (*E*)-2-hexenal (de Bruyne et al., 2001), although ab4A is also very sensitive to other ligands, including hexanal (Figure S6). Contrary to ab4B, ab4A houses only one OR, namely, DmelOr7a (Hallem et al., 2004). Because expression of CquiOR32 was driven by DmelOrco, ab4A neurons in our transgenic flies house both DmelOr7a and CquiOR32. Coexpression was confirmed by a significantly stronger response to methyl salicylate (p = 0.0001, unpaired t test) recorded from Orco-Gal4/UAS-CquiOR32 than from WT flies (Figures 7 and S6), while retaining response to hexanal (Figure S7). It is known that methyl salicylate is the best ligand for DmelOr10a in ab1D (Hallem et al., 2004) but elicits only very low response in ab4A (de Bruyne et al., 2001). The low response of WT flies to methyl salicylate did not differ significantly (p > 0.9999) when the odorant was delivered alone or codelivered with eucalyptol (Figure 7). By contrast, responses recorded from Orco-Gal4/UAS-CquiOR32 flies were significantly lower (p = 0.0003) when the two stimuli were delivered simultaneously from two different cartridges (Figures 7 and S6). Next, we tested whether CquiOR32 response to eucalyptol would affect DmelOR7a response to a cognate ligand, hexanal. Responses of WT flies to hexanal did not differ significantly when comparing hexanal alone with hexanal plus eucalyptol (Figures S7 and S8). Recordings from ab4 sensilla in the Orco-Gal4/UAS-CquiOR32 flies showed a slight, albeit not significant, increase in response to hexanal. This is unlikely to be due to hexanal activation of CquiOR32 (Table S1, entry #128). When hexanal and eucalyptol were delivered simultaneously firing of DmelOR7a was completely abolished (Figures S7 and S8). We also recorded from ab7 sensilla, which expresses DmelOR98a, in ab7A and for which butyl acetate is one of the best ligands (Munch and Galizia, 2016). Eucalyptol elicited inhibitory response in ab7A neurons of Orco-Gal4/UAS-CquiOR32 flies (Figure S9). In the transgenic flies both methyl salicylate and butyl acetate generated excitatory responses (Figure S9), which were inhibited by eucalyptol (Figures 8 and S9). Because methyl salicylate and eucalyptol elicit inward and reverse currents in CquiOR32, this *in vivo* inhibition is not surprising. However, the consistent observation that eucalyptol inhibits the response of an endogenous receptor to a cognate ligand supports the notion that intraneuronal inhibition occurs when receptors are colocated in a neuron. Specifically, the inhibitory responses of CquiOR32 interferes with the activation of a collocated receptor by a cognate ligand. For example, activation of DmelOR7a in ab4A neuron by hexanal and activation of DmelOR98a in ab7A neuron by butyl acetate were both inhibited by eucalyptol upon interaction with CquiOR32. Contrary to the fruit fly, which expresses only one receptor per neuron (with a few exceptions), mosquitoes (and possibly other insect species) can coexpress multiple ORs in the same neuron (Karner et al., 2015).

### Inhibition in *Culex* Mosquito Antennae

**Figure 9:**
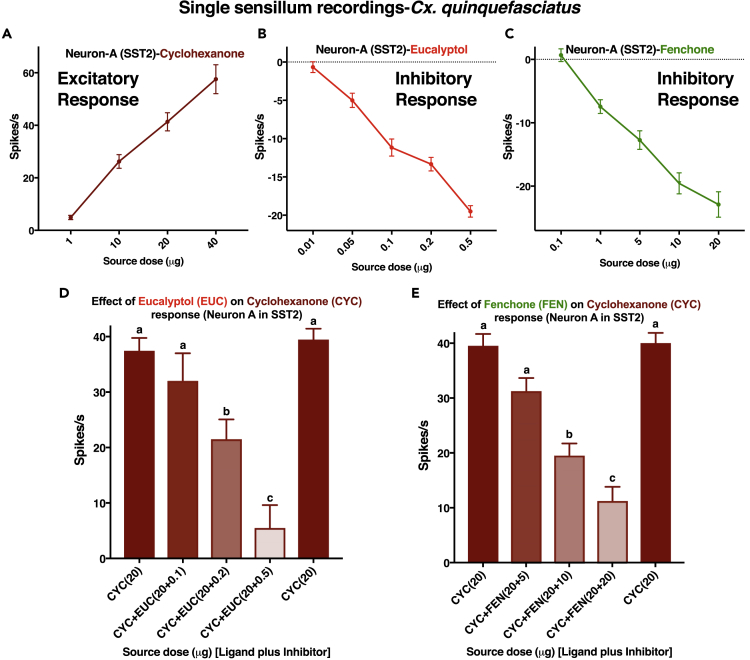
Single Sensillum Recordings from Short Sharp-Tipped-2 (SST-2) Sensilla on *Cx. quinquefasciatus* Antennae (A–C) Dose-dependent curves for excitatory and inhibitory compounds: (A) cyclohexanone, (B) eucalyptol, and (C) fenchone. (D and E) Effect of eucalyptol (EUC) (D) and fenchone (FEN) (E) on the excitatory responses elicited by cyclohexanone (CYC). Stimuli were delivered in sequence as displayed from left to right. Firing rates observed during 500 ms post-stimulus period were subtracted from spontaneous activities observed in the 500 ms pre-stimulus period, and the outcome was multiplied by 2 to obtain the number of spikes per second. The recording points in x axis are not drawn to scale. Error bars represent SEM. n = 6–12. Bars with different letters are considered statistically different at the 0.05 level, according to Tukey's test.

We investigated by using SSR whether inhibition would occur in antennae of the southern house mosquito. Of 240 contacts, 33 recordings from ORN-A in short sharp-tipped sensilla 2 (SST-2) showed that this neuron responded to cyclohexanone with dose-dependent excitatory responses (Figure 9A) and to eucalyptol (Figure 9B) and fenchone (Figure 9C) with dose-dependent inhibitory responses, thus resembling what has been observed in the *Xenopus* oocyte recording system (Figure 1). Additionally, the responses to cyclohexanone were modulated by both eucalyptol (Figure 9D) and fenchone (Figure 9E) in a dose-dependent manner.

### CquiOR32 Orthologue in *Aedes aegypti* and Intrareceptor Inhibition

CquiOR32 has an orthologue in the genome of the yellow fever mosquito, AaegOR71 (AAEL017564), with 55.5% identity. We sequenced 20 clones and obtained 19 AaegOR71 sequences. Five clones showed sequences identical to the sequence in VectorBase and were, therefore, considered the WT. We expressed AaegOR71-WT in the *Xenopus* oocyte recording system (along with AaegOrco) and challenged the oocytes with compounds that elicited inward and inhibitory currents in CquiOR32. Cyclohexanone elicited inward currents, but the compounds generating the largest inward currents were 4,5-dimethylthiazole (DMT) and 2-methyl-2-thiazoline (2MT) (Figure S10); no response was observed with methyl salicylate. Although eucalyptol and fenchone did not elicit measurable inhibitory currents, these two compounds reduced AaegOR71 responses to cyclohexanone, DMT, and 2MT (Figure S10).

Four clones (AaegOR71-V2, GenBank MG593069) differed from WT in 7 amino acid residues, and 3 clones (AaegOR71-V1, MG593068) differed from WT in 11 amino acid residues. They both showed weak responses to odorants when tested in the *Xenopus* oocyte recording system. The other 7 clones differed from the WT in 6–12 amino acid residues. AaegOR71-V5 (MG593071), AaegOR71-V14 (MG593074), and AaegOR71-V15 (MG593075) differed in 12, 9, and 11 amino acid residues, respectively, and none of them responded to odorants. AaegOR71-V8 (MG593072) differed in 10 amino acid residues and showed very weak response only to the Orco agonist VUAA-1. AaegOR71-V4 (MG593070), AaegOR71-V9 (MG593073), AaegOR71-V17 (MG593076) differed in 8, 10, and 6 amino acid residues but gave weak to moderate responses to odorants. None of these variants elicited detectable inhibitory currents when challenged with eucalyptol or fenchone.

### Peripheral Inhibition in the Yellow Fever Mosquito Antennae

**Figure 10:**
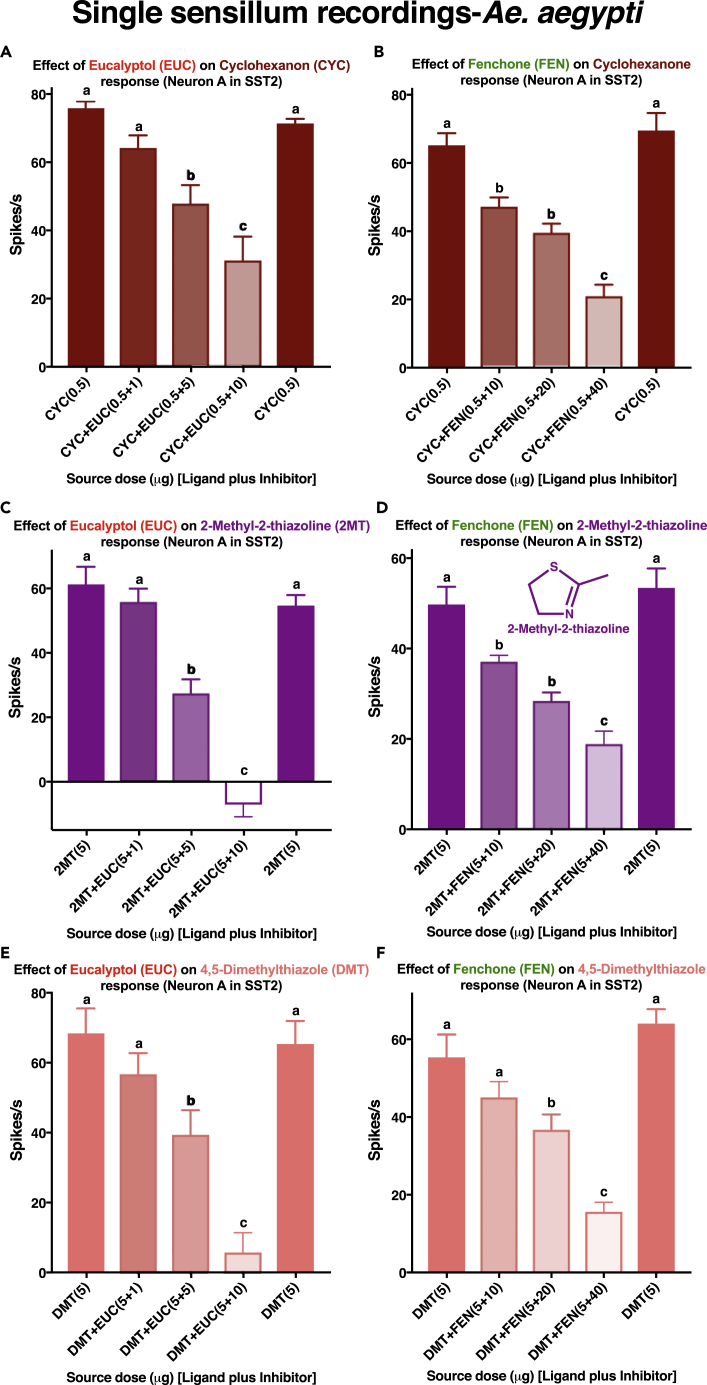
Quantification of Responses from Neuron-A in the Short Sharp-Tipped-2 (SST-2) Sensilla on *Ae. aegypti* Antennae The sensilla were challenges with mixtures of (A and B) cyclohexanone, (C and D) 2-methyl-2-thiazoline, or (E and F) 4,5-dimethylthiazole in combination with either eucalyptol (A, C, and E) or fenchone (B, D, and F). Stimuli were delivered in sequence as displayed from left to right. Firing rates observed during 500 ms post-stimulus period were subtracted from spontaneous activities observed in the 500 ms pre-stimulus period, and the outcome was multiplied by 2 to obtain the number of spikes per second. Error bars represent SEM. n = 6.

Next, we tested whether intrareceptor inhibition might be manifested *in vivo* in the antennae of the yellow fever mosquito. With 350 contacts, 69 recordings were made from SST-2 sensilla. SSR showed that cyclohexanone, 2-methyl-2-thiazoline, and 2,4-dimethylthiazole elicited dose-dependent excitatory responses in neuron-A in SST-2, whereas eucalyptol and fenchone showed inhibitory responses (Figure S11). When costimulated with 2-methyl-2-thiazoline and eucalyptol, the response to the odorant decreased markedly (Figure S12). We then analyzed the effect of inhibitors on the responses to the three odorants that caused excitatory responses. Both eucalyptol and fenchone inhibited the responses of ORN-A in SST-2 to cyclohexanone (Figures 10A and 10B), 2-methyl-2-thiazoline (Figures 10C and 10D), and 4,5-dimethylthiazole (Figures 10E and 10F) in a dose-dependent manner.

### Intrareceptor Inhibition Manifested in Mosquito Behavior

**Figure 11:**
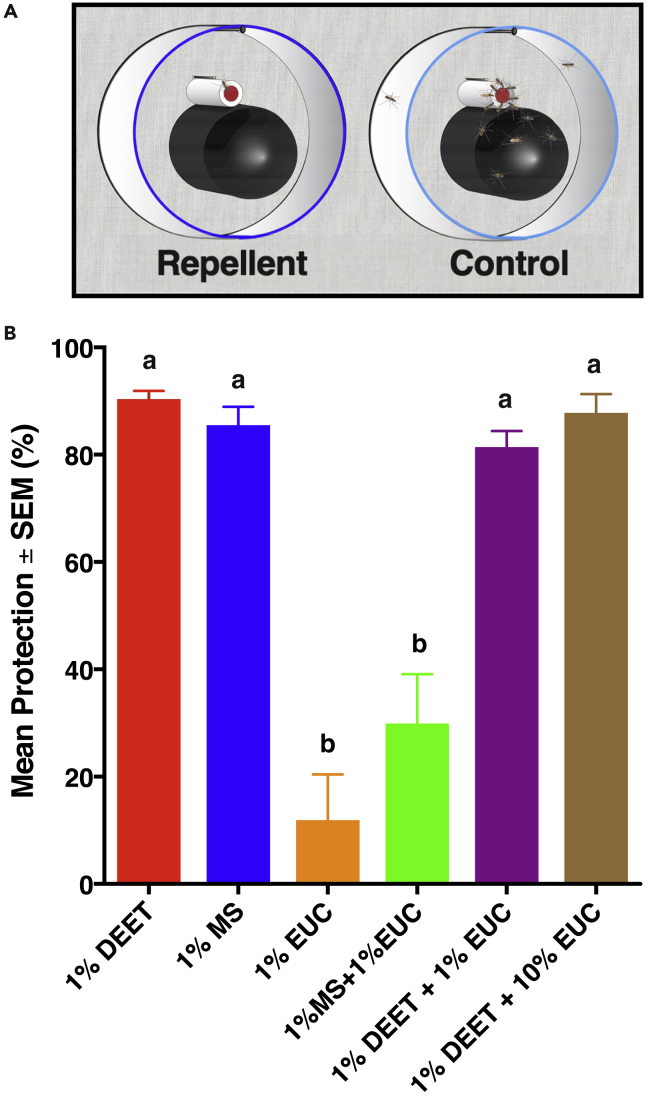
Effect of Eucalyptol on Methyl Salicylate-Elicited Repellency (A) Diagram of the surface landing and feeding assay, (B) protection data. Eucalyptol (EUC) at 1% showed no repellency, but methyl salicylate (MS) or DEET at 1% repelled blood-seeking *Culex* mosquitoes. MS-elicited repellency was significantly reduced when tested in a mixture of 1% MS and 1% EUC, but EUC at 1% or 10% had no effect on DEET repellency. Error bars represent SEM. n for each bar from left to right: 35, 16, 12, 31, 28, and 11, respectively.

Evidence in the literature suggests that eucalyptol is an oviposition deterrent (Klocke et al., 1987). We then tested in our surface landing and feeding assay (Leal et al., 2017) (Figure 11A) whether this inhibitory compound or other odorants would repel blood-seeking *Cx. quinquefasciatus* mosquitoes. In preliminary experiments, eucalyptol at 0.1%, 47.57 ± 3.15% mosquitoes responded to the treatment side of the arena, whereas 52.43 ± 3.15% responded to the control (n = 10, p = 0.3276; eucalyptol, 7.5 ± 0.4 mosquitoes in treatment and 8.5 ± 0.8 mosquitoes in the control side). With a higher dose (1%), 44.3 ± 5.7% and 55.7 ± 5.7% mosquitoes responded to treatment and control, respectively (n = 10, p = 0.3330; treatment, 8.1 ± 1.1; control 10.1 ± 1.0 mosquitoes), thus showing that eucalyptol per se is not a potent spatial repellent. Likewise, cyclohexanone did not show repellence activity at 0.1% (47.7 ± 4.2% treatment versus 52.3 ± 4.2% control, n = 4, p = 0.7027; 7 ± 1.1 and 7.5 ± 0.5 in treatment and control, respectively) or 1% (52.7 ± 2.7% treatment versus 47.3 ± 2.7% control, n = 4, p = 0.4950; 8.25 ± 0.9 and 7.75 ± 1.4 in treatment and control, respectively).

Contrary to eucalyptol, methyl salicylate showed repellence activity. At a dose of 0.1%, the number of mosquitoes responding to the treatment side of the arena was significantly lower than the number responding to control (39.9 ± 4.0% treatment versus 60.1 ± 4.0% control, n = 10, p = 0.0303; 8 ± 0.8 and 12.3 ± 1.1 in treatment and control, respectively). At a higher dose (1%), the difference was highly significant (15.3 ± 3.7% treatment versus 84.5 ± 3.7% control, n = 10, p < 0.0001; 1.9 ± 0.8 and 8.3 ± 0.9 in treatment and control, respectively).

Because eucalyptol per se is not a repellent, we then tested the effect of eucalyptol on repellence elicited by methyl salicylate. A mixture of 1% methyl salicylate and 1% eucalyptol had significantly lower protection than methyl salicylate alone (Video S1). We repeated these experiments and tested whether eucalyptol would inhibit DEET repellency (Figure 11B). Here we expressed the results in protection so as to infer the potency of each repellent alone as well as in combination with eucalyptol (Figure 11B). Again, eucalyptol at 1% showed no protection, whereas methyl salicylate and DEET at 1% had comparable protection when freshly applied. Addition of eucalyptol at 1% to methyl salicylate led to a loss of activity. By contrast, DEET repellency was not affected when DEET 1% was mixed with eucalyptol either at 1% or 10% (Figure 11B). These behavioral measurements suggest that eucalyptol modulates the mosquito olfactory response to methyl salicylate (but not DEET), thus reducing the behavioral response. Eucalyptol showed no effect on DEET, which is detected by CquiOR136 (Xu et al., 2014). These findings support the notion that the above-described inhibition is manifested in mosquito behavior.

Video S1. Repellency Activity Elicited by Methyl Salicylate Is Reduced in the Presence of Eucalyptol, Related to Figure 11

### Conclusions

We provided evidence showing that the same receptor (CquiOR32) is activated by some ligands and inhibited by others (intrareceptor inhibition). When this “inhibitory receptor” was coexpressed with another OR we found evidence of intraneuron inhibition. Are these inhibitory compounds merely inverse agonists? If receptors are constitutively open as previously suggested for a case in the vinegar fly (Cao et al., 2017), binding to eucalyptol (or another inhibitor) could shift the equilibrium toward the closed, inactive form of the receptor. However, CquiOR32 agonist and inhibitors are chemically so dissimilar that is hard to conceive, albeit possible, that both types of compound bind to the same orthosteric binding site as in the case of inverse agonists. It is, therefore, more likely that these are negative allosteric modulators. Of note, there is already evidence in the literature suggesting that there is positive allosteric modulation of mosquito ORs when expressed in insect cells (Tsitoura and Iatrou, 2016). Here we found *in vitro* and *in vivo* evidence suggesting that negative allosteric modulation may occur. It is known that insect ORx-Orco complexes form agonist-activated cation channels (Wicher et al., 2008, Smart et al., 2008, Sato et al., 2008). Upon binding, cation channels are open and an influx of cations generates inward currents. Theoretically, outward currents could be elicited by chloride influx. In insects, the difference in chemical composition of the sensillum (=receptor) lymph and hemolymph generates the standing electrical potential, i.e., the transepithelial potential, and it favors a possible Cl^−^ influx (Steinbrecht, 1992): sensillum lymph, K^+^ 200 mM; Na^+^ 25 mM; Cl^−^, 25 mM; hemolymph, K^+^, 36 mM; Na^+^ 12 mM; Cl^−^, 12mM; organic anions make the balance (Kaissling and Thorson, 1980). In the mammalian olfactory system in which the Cl^−^ gradient is reversed, Cl^−^ efflux (inward current) depolarizes the membrane further (Hartzell et al., 2005), thus providing the most pronounced amplification of the odor signal within the whole signal transduction cascade (Wicher, 2015). In summary, the exact mode of action of these putative negative allosteric modulators are yet to be elucidated, but apparently Cl^−^ currents are involved.

### Limitations of the Study

Our dataset provided strong evidence for intrareceptor and intraneuron inhibition, but to unambiguously elucidate the mode of action of these inhibitory responses that lead to (outward) reverse currents in oocytes and “inverse” EAG responses we must wait for the elucidation of the structures of CquiOR-CquiOrco or other OR-Orco complexes with similar features.

## Methods

All methods can be found in the accompanying Transparent Methods supplemental file.

## Supplemental Information

Document S1. Transparent Methods and Figures S1–S12

Table S1. Tested Compounds and Their Activities, Related to Figure 1
