# Supplementary material for: Odorant Inhibition in Mosquito Olfaction
Source: iScience. 2019 Jul 12;19:25–38. doi: 10.1016/j.isci.2019.07.008 (PMC6660600; doi:10.1016/j.isci.2019.07.008)
Supplement: Document S1. Transparent Methods and Figures S1–S12 [file mmc1.pdf]

ISCI, Volume *19*

Supplemental Information

Odorant Inhibition in Mosquito Olfaction

Pingxi Xu, Young-Moo Choo, Zhou Chen, Fangfang Zeng, Kaiming Tan, Tsung-Yu Chen, Anthony J. Cornel, Nannan Liu, and Walter S. Leal

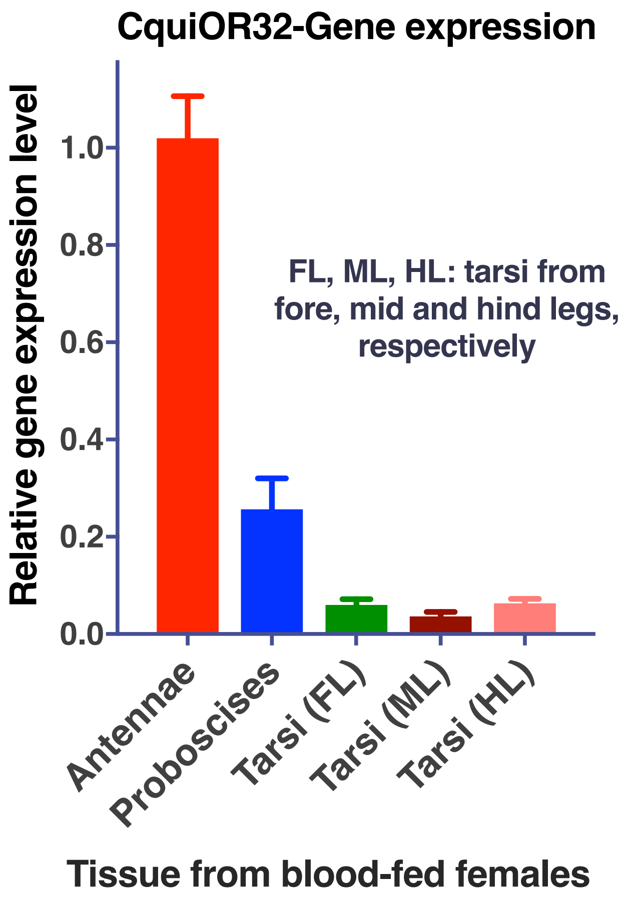

Figure S1. Quantitative PCR data for CquiOR32. This receptor is predominantly expressed in female antennae. Error bars represent SEM. n = 3 biological samples, each with 3 biological replicates.

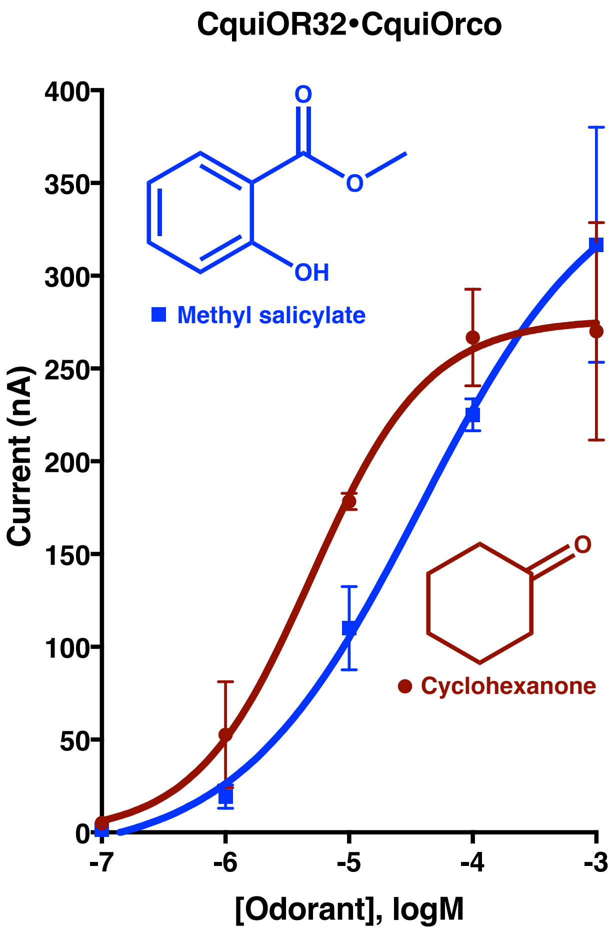

Figure S2. Concentration-response relationships, Related to Figure 1. EC_{50} for methyl salicylate and cyclohexanone: $3.7X10^{-5}$M and $4.9X10^{-6}$M, respectively. N = 3 for each data point, each from a different oocyte of the same batch of eggs. Error bars represent SEM.

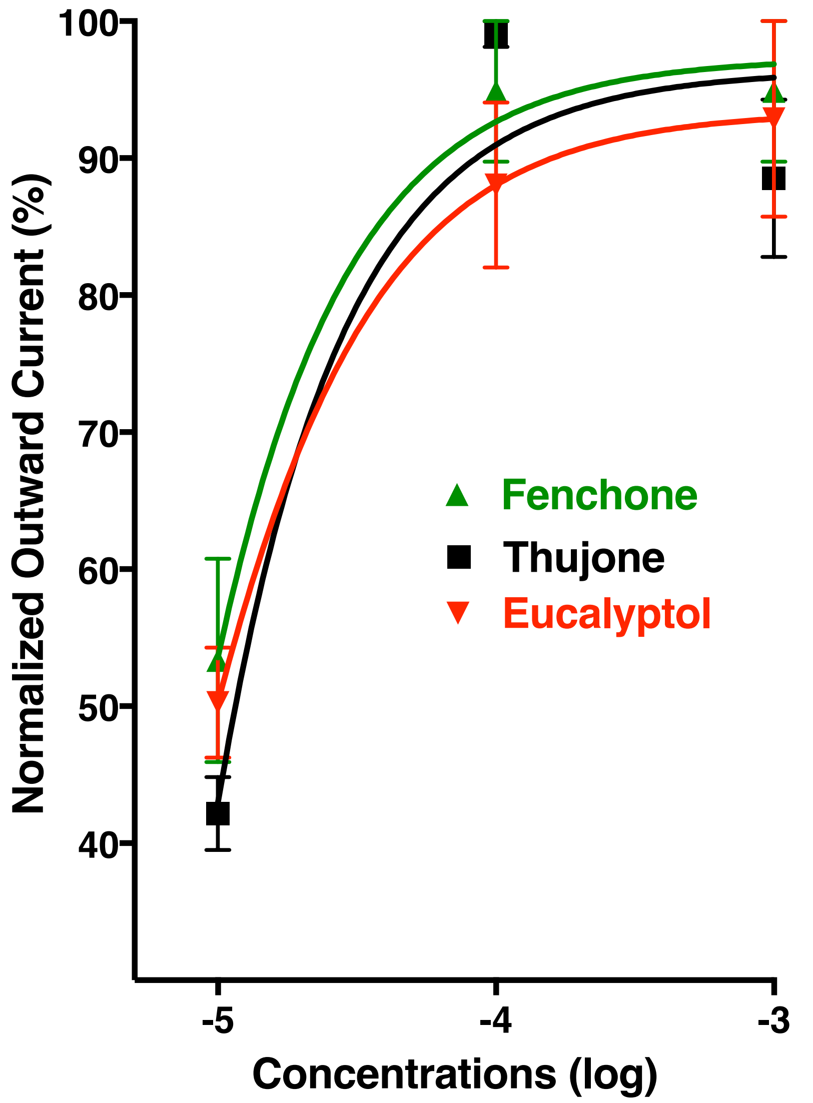

Figure S3. Dose-dependence curves for inhibitory compounds, Related to Figure 1.

Responses were normalized (n = 3 for each compound and dose). Error bars represent SEM.

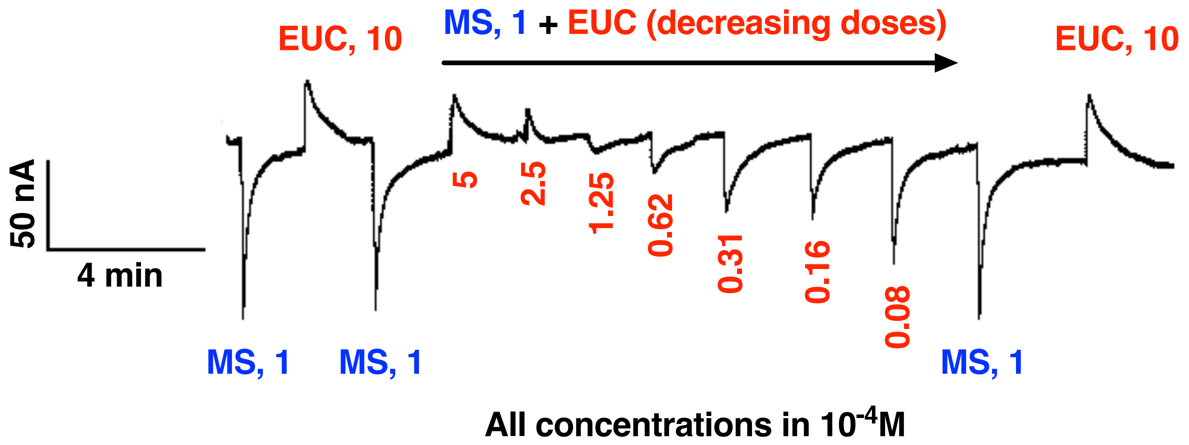

Figure S4. **Eucalyptol (EUC)-elicited, dose-dependent inhibition of responses of CquiOR32-CquiOrco-expressing oocytes to methyl salicylate (MS).** Continuous trace from the same oocyte.

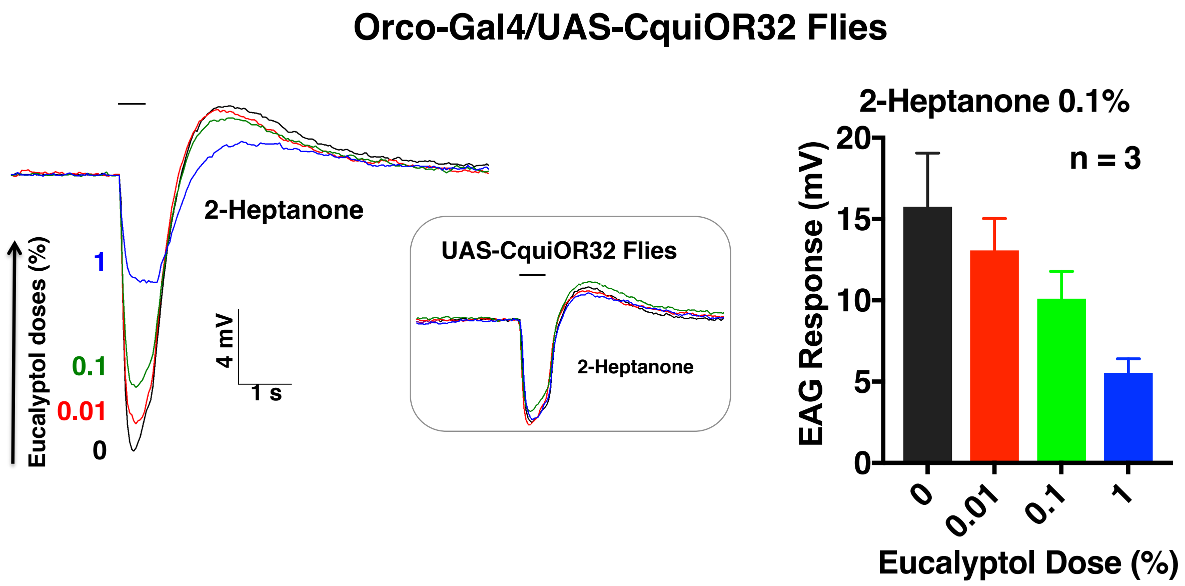

Figure S5. Inhibitory effect of eucalyptol on odorant reception in transgenic flies, Related to Figure 6. Odorant (0.1%) was codelivered onto fly antennae with eucalyptol at various doses. Error bars represent SEM. *Inset:* EAG recordings from a control line stimulated with 2-heptanone alone or coapplied with eucalyptol (0.01-1%).

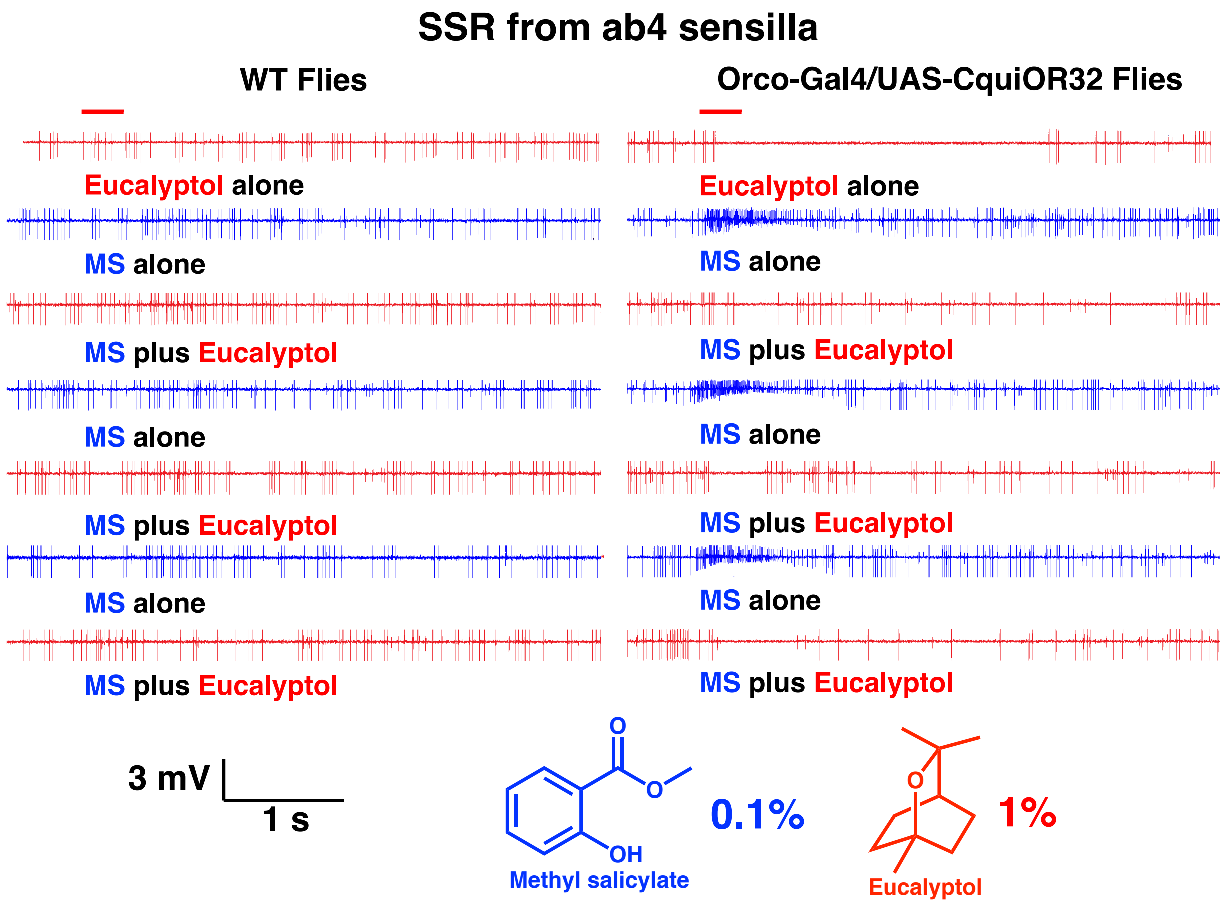

Figure S6. **Single sensillum recordings from the ab4 sensilla in the antennae of WT and transgenic flies, Related to Figures 6 and 7.** Traces obtained by challenging the sensilla with methyl salicylate (MS) at 0.1% and eucalyptol at 1%.

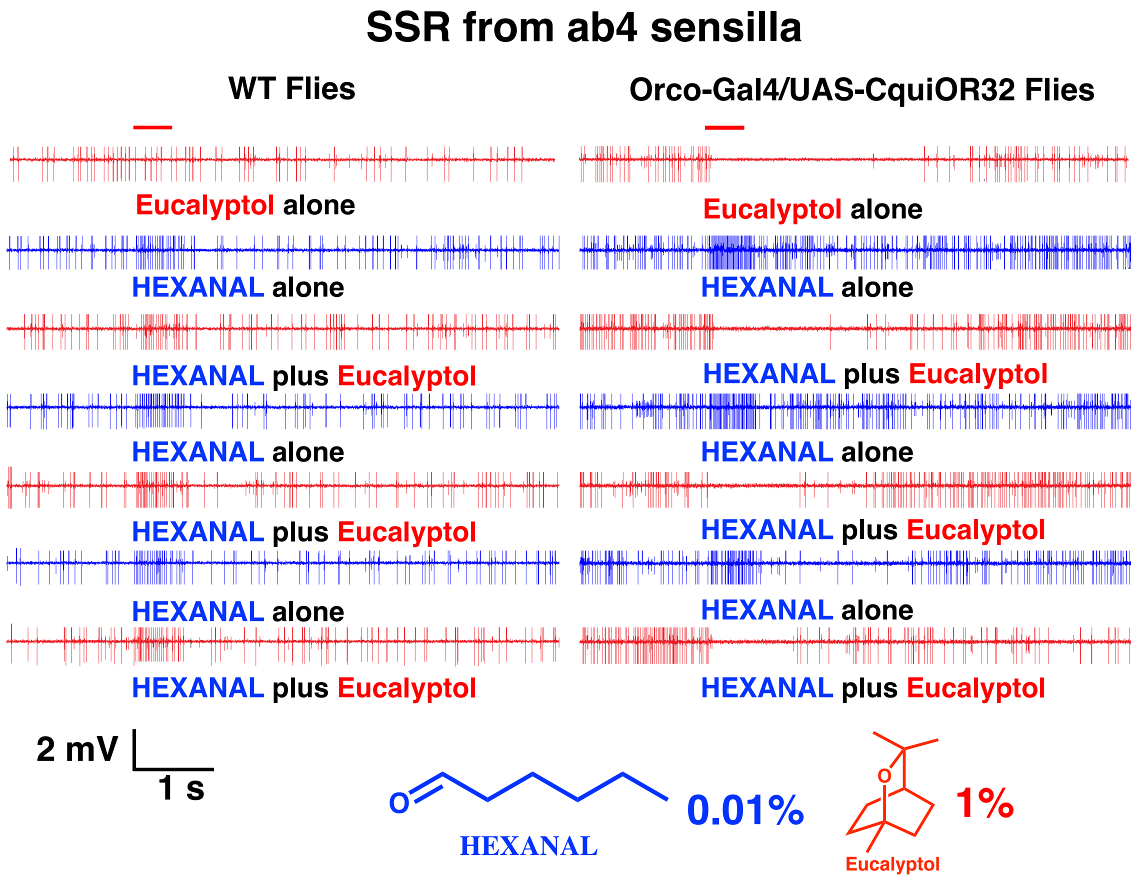

Figure S7. Single sensillum recordings from the ab4 sensilla in the antennae of WT and transgenic flies, Related to Figure 6. Traces obtained by challenging the sensilla with hexanal at 0.01% and eucalyptol at 1%.

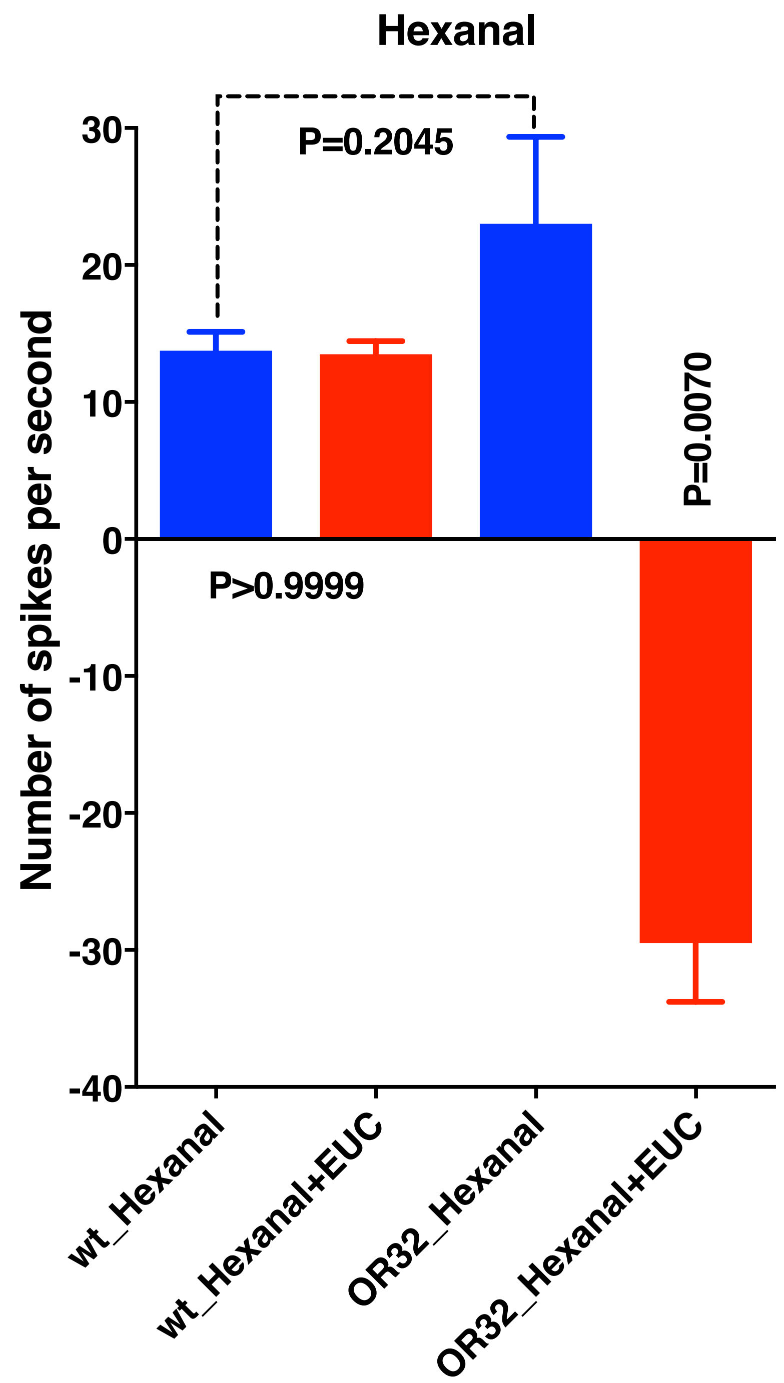

Figure S8. Quantification of responses of WT and transgenic flies to hexanal alone or in combination with eucalyptol, Related to Figure 6. Costimulation with eucalyptol (EUC) did not affect the response of WT flies to hexanal. By contrast, the response of Orco-GAL4/UAS-CquiOR32 flies to hexanal was completely abolished by costimulation with eucalyptol.

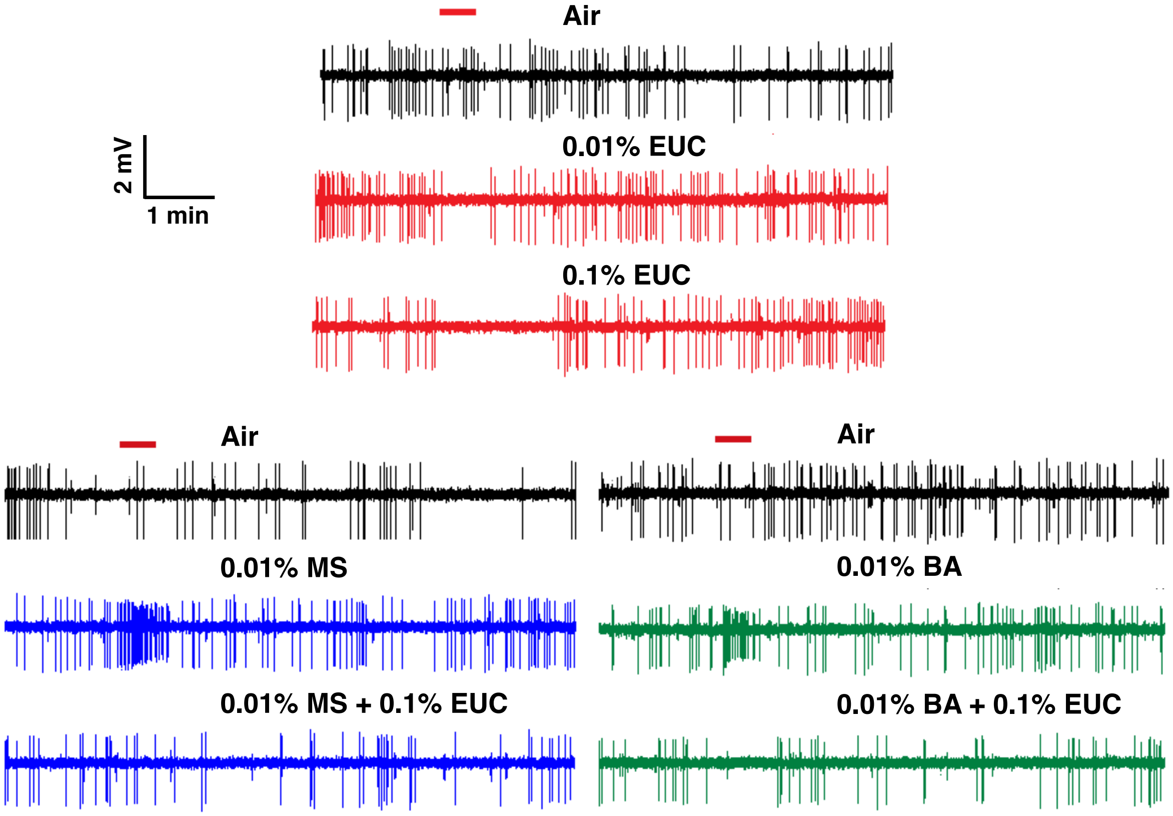

Figure S9. **Single sensillum recordings from the ab7 sensilla in the antennae of Orco-GAL4/UAS-CqOR32 flies, Related to Figure 8.** Inhibitory responses elicited by 0.01 and 0.1% eucalyptol (EUC), excitatory responses generated by 0.01% methyl salicylate (MS) or 0.01% butyl acetate (BA), and inhibition caused by costimulation with 0.1% EUC.

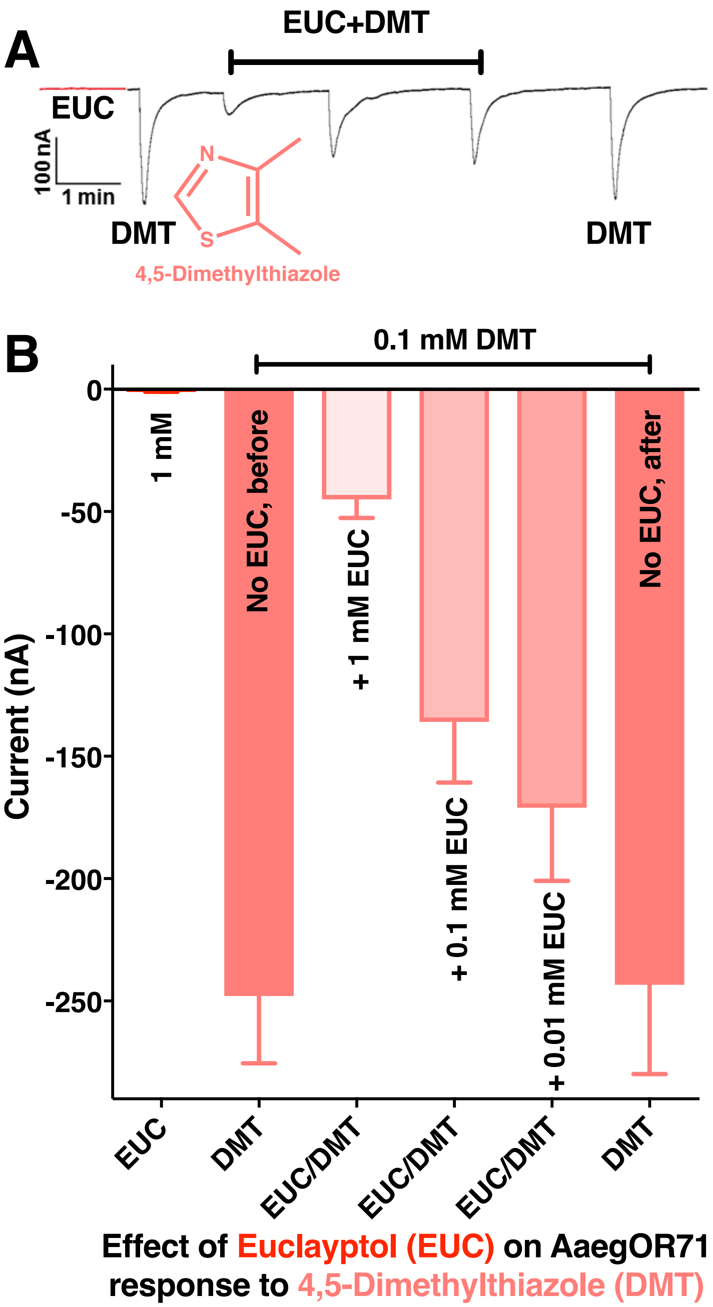

Figure S10. Eucalyptol inhibition of responses elicited by 4,5-dimethylthiazole (DMT) on oocytes coexpressing AaegOR71 and AaegOrco, Related to Figure 9. Although eucalyptol (EUC) did not elicit detectable inhibitory currents, it caused a dose-dependence reduction in DMT-elicited responses. Error bars represent SEM. n = 5. Bars with different letters are considered statistically different at the 0.05 level, according to Tukey's test.

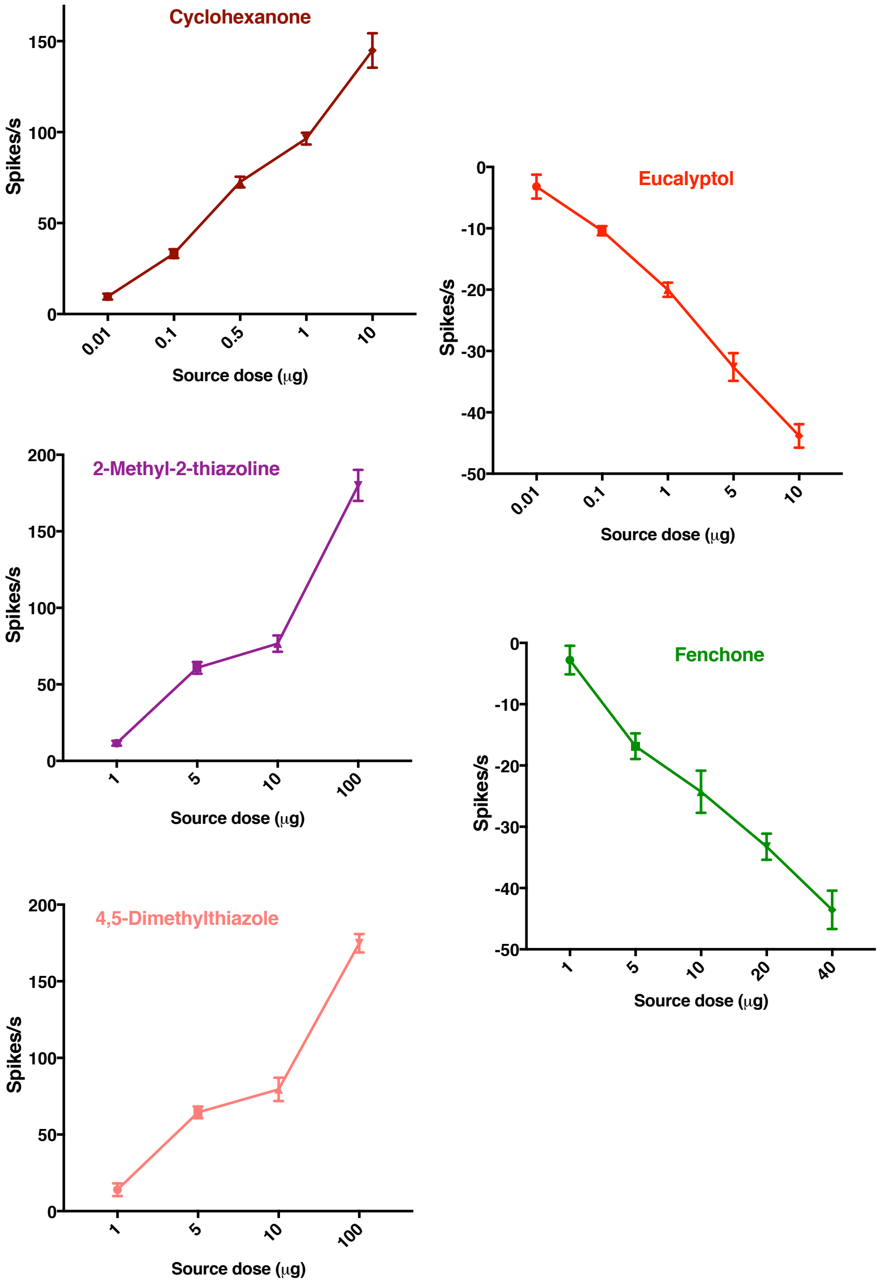

Figure S11. **Dose-dependent curves recorded from *Ae. aegypti* antennae by SSR, Related to Figure 10.** Firing rates observed during 500 ms post-stimulus period were subtracted from spontaneous activities observed in the 500 ms pre-stimulus period and the outcome was multiplied by 2 to obtain the number of spikes per second. The recording points in X-axis are not drawn to scale. Error bars represent SEM. n = 5-20.

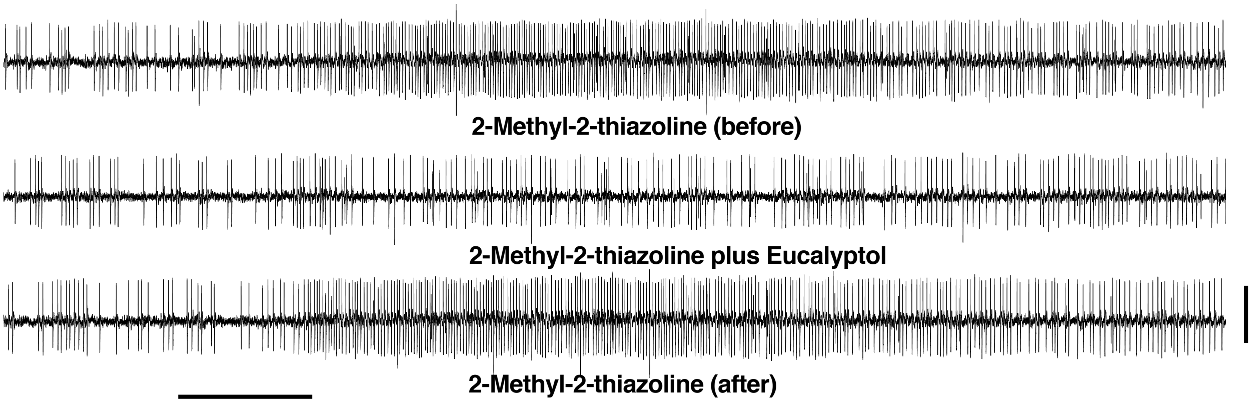

Figure S12. **SSR traces from SST-2 sensilla on *Ae. aegypti* antennae, Related to Figure 10.**

A bar beneath the traces indicates the duration of the stimulus (500 ms). Horizontal line after the bottom trace, 4 mV.

Transparent Methods

Insect preparations

Cx. quinquefasciatus used in this study were from a laboratory colony originating from adult mosquitoes collected in Merced, CA in the 1950s (Syed and Leal, 2008) and kept at the Kearney Agricultural Research Center, University of California, Parlier, CA. Specifically, we used mosquitoes from the UC Davis colony, which was initiated about 7 years ago with mosquitoes from the Kearney colony. In Davis, mosquitoes were maintained at 27±1°C, 75±5% relative humidity, and under a photoperiod of 12:12 h. Two colonies of *Ae. aegypti* were used in this study, namely, the Orlando strain, kept at Auburn University, and a colony established in 2016 from eggs that were laid by females collected in BG-sentinel traps (Biogents, Regensburgs, Germany) in the City of Clovis, California. These colonies were maintained at 25±2°C, 75±5% relative humidity, and under a photoperiod of 12:12 h.

Behavioral studies

Repellency was measured using a previously reported surface landing and feeding assay (Leal et al., 2017). The arena (30.5 × 30.5 × 30.5 cm) housing test mosquitoes was attached to a frame that supported a wood board (30 × 30 × 2.5 cm) and held two Dudley bubbling tubes and two syringe needles separated from each other by 17 cm. The Dudley tubes were painted internally with black glass ink and dried before use. They were placed on a transverse plane at the middle line of the wooden board. One side of the mosquito cage was prepared with a red cardstock having openings to allow the Dudley tubes and syringe needles to protrude inside of the mosquito cage by 5.5 and 4 cm, respectively. Each syringe was placed 8 mm above a

Dudley tube so to have enough room to tightly hold dental cotton rolls. Insect pins were placed 1.8 cm above the syringe needles to hold filter paper rings (width 4 cm; 25 cm; overlapped 1 cm for stapling), which were loaded with test repellent or solvent (hexane, control). For tests with two compounds (eg, methyl salicylate and eucalyptol), each compound was applied to one filter paper ring and the two rings were placed concentrically in the test side of the arena. Once defibrinated sheep blood (100 μl) was loaded on dental cotton rolls, carbon dioxide started to flow at 50 ml/min from each needle, and water at 38 °C circulated inside the Dudley tubes. The two choices differed only by a curtain of repellent at the side with the treatment filter paper ring. Assays were recorded with a camcorder equipped with Super NightShot Plus infrared system (Sony Digital Handycam, DCR-DVD 810) and the number of mosquitoes on each side of the arena were counted at the end of each test. Behavioral responses are expressed in protection rate, according to WHO and EPA recommendations: P%=(1-[T/C]) X 100, where T and C represent the number of mosquitoes in treatment and control sides of the arena.

OR cloning

Total RNA samples were extracted from 1 thousand 4-7 day-old *Culex* female antennae and 800 *Aedes* female antennae with TRIzol reagent (Invitrogen, Carlsbad, CA). Antennal cDNA was synthesized from 1 μg of antennal total RNA from each species using a SMARTer™ RACE cDNA amplification kit according to manufacturer's instructions (Clontech, Mountain View, CA). To obtain full-length coding sequences of CquiOR32, PCRs were performed using gene-specific primers containing restriction endonuclease sites and Kozak motif (acc): CquiOR32 Fwd-XmaI (underlined) primer, 5'-

TCCCCCCGGGGGAaccATGTTCACCACTCAAAACAGTCACC-3'and Rev-XbaI (underlined) primer, 5'-GCTCTAGAGCTTATATCATAGTCAACGCTTCCTTCAGCA-3'. PCR products were purified by a QIAquick gel extraction kit (Qiagen) and then cloned into pGEM-T vector (Promega). Plasmids were extracted using a QIAprep spin mini prep kit (Qiagen) and sequenced (Davis Sequencing). To subclone CqOR32 into pGEMHE, pGEM-T-CquiOR32 was digested by XmaI and XbaI (BioLabs) before being subcloned. After transformation, plasmids were extracted using the QIAprep Spin Miniprep kit (Qiagen) and sequenced by ABI 3730 automated DNA sequencer at Davis Sequencing (Davis, CA) for confirmation. Likewise, AaedOR71 was cloned with a pair of cloning primers: AaeagOR71-Fwd: AGATCAATTCCCCGGGaccATGGAACTGTCCTACCATCGAAGTC

AaegOR71-Rev: TCAAGCTTGCTCTAGACTAAAGCCGATCCAGAACATCTTTG

Quantitative PCR, qPCR.

For qRT-PCR, each type of tissue (antennae, maxillary palps, proboscis, and legs) from 300 blood-fed female mosquitoes (4-7 days old) was dissected and collected in TRIzol reagent (Invitrogen, Carlsbad, CA) on ice using a stereo microscope (Zeiss, Stemi DR 1663, Germany). Total RNA was extracted using TRIzol reagent. After RNA was quantified on NanoDrop Lite spectrometer (Thermo Fisher Scientific, Rockford, IL), cDNA was synthesized from 200 ng of equal amount RNA using iScript™ Reverse Transcription Supermix for RT-qPCR according to the manufacturer's instructions (Bio-Rad, Hercules, CA). Real-time quantitative PCR (qPCR) was carried out by using a CFX96 Touch^{TM} Real-Time PCR Detection System (Bio-Rad) and SsoAdvanced SYBR Green Supermix (Bio-Rad). *CquiRPS7* gene was used as the reference. The following primers, designed by Primer 3 program (http://frodo.wi.mit.edu/), were used:

CquiOR32-Fw: 5'-GCGATTTTTGCTTCGAAAAG-3'

CquiOR32-Rv: 5'-GTGCGTCCAATACCGAAAGT-3'.

qPCR was performed with 3 biological replicates, and each of them was replicated 3 times (3 technical replicates per biological replicate); data were analyzed using the $2^{-\Delta\Delta CT}$ method.

Electrophysiology

D. melanogaster w^{1118} and Orco-GAL4/UAS-CquiOR32 homozygous flies were used for EAG (Ueira-Vieira et al., 2014) and other electrophysiological studies. The EAG apparatus (Syntech Ltd., Hilversum, The Netherlands) was linked to a computer with an EAG2000 data acquisition interface. Recording and indifferent electrodes were made of Ag/AgCl wires enclosed in drawn glass capillary needles, which were filled with 1 M potassium chloride in 1% polyvinylpyrrolidone. The reference electrode was inserted in the eye of an immobilized insect and the recording electrode was placed on the third segment of a fruit fly antenna by using a micromanipulator MP-12 (Syntech). Single sensillum recording from flies were obtained as previously reported (Syed et al., 2010). Compounds used as stimuli were freshly dissolved in paraffin oil and loaded on a filter paper strip (1 cm^2), which were placed into Pasteur pipettes as cartridges. The preparation was bathed in a high-humidity air stream flowing from a Stimulus Controller CS-55 (Syntech) at 610 mL/min to which compensatory flow or stimulus pulse (125 mL/min, 300 ms) was added. For dual delivery, the stimulus flow was split and passed through 2 cartridges, each one having a filter paper strip laden with one of the tested compounds. The outlets of these cartridges merged in the continuous flow and placed 1 cm away from the antennal preparation. Signal from the antenna induced by stimulus or control puff was recorded for 10 s. Gas chromatography with electroantennographic detection (GC-

EAD) was done with a gas chromatograph (HP 5890 Series II Plus, Agilent Technologies, Palo Alto, CA) equipped with transfer line and temperature control units (Syntech, Kirchzarten, Germany). The effluent from the capillary column was split into EAD and flame ionization detector (FID) in a 3:1 ratio. The analog signal was fed into an A/D 35900E interface (Agilent Technologies) and acquired simultaneously with FID signal on an Agilent Chemstation. The gas chromatograph was equipped with a capillary column (HP-5MS, 30 m×0.25 mm; 0.25 µm; Agilent Technologies). The temperature program started at 70°C for 1 min, increased at a rate of 10°C/min to 110°C then increased at a rate of 20°C/min to 290°C and finally held at this final temperature for 1 min. After identifying the retention times of eucalyptol and methyl salicylate, data were acquired from 4 to 8 min. Both injection port and detector were operated at 250ºC.

For mosquito SSR, 4- to 5-day-old females were used after being anesthetized on ice and fixed with a 200-µL pipette tip (6). Mosquitoes were fixed with dental wax and using a cover slip (22 X 22 mm) with double-sided tape. The reference tungsten electrode was inserted into one eye of a test mosquito, whereas the recording electrode was inserted into the shaft of a test sensillum under a microscope (Leica Z6 Apo) by using a micromanipulator (Leica, Cat #: 115378). Chemical compounds used as stimuli were freshly prepared with dimethyl sulfoxide (DMSO) at desired concentrations and 1 µL of each chemical solution was dispersed onto a piece of filter paper (3 × 45 mm), which was inserted into a glass Pasteur pipette to create a stimulus cartridge. The preparation was bathed in a high-humidity air stream flowing from a Stimulus Controller CS-55 (Syntech) at 20 mL/s to which compensatory flow or stimulus pulse (0.5 L/min, 500 ms) was added. The signal acquired by a preamplifier (Universal AC/DC Probe Gain 10X, Syntech) was digitized by using an IDAC 4 (Syntech). Action potential evoked by stimulus or control puff was recorded for 10 s, starting 1 s before the stimulation.

Action potentials were counted off-line over a 500-ms period before and after the stimulation (Liu et al., 2013). Specifically, firing rates observed during 500 ms post-stimulus were subtracted from spontaneous activities observed in the 500 ms pre-stimulus period and the outcome was multiplied by 2 to obtain the number of spikes per second (Liu et al., 2013).

The 2-electrode voltage-clamp technique (TEVC) was performed as previously described (Leal et al., 2013, Xu et al., 2014, Xu et al., 2012a, Xu et al., 2012b, Zhu et al., 2013). Briefly, the capped cRNAs were synthesized using pGEMHE vectors and mMESSAGE mMACHINE T7 Kit (Ambion). Purified OR cRNAs were resuspended in nuclease-free water at 200 ng/mL and 9.2 nl aliquots were microinjected with the same amount of CquiOrco cRNA into *Xenopus laevis* oocytes in stage V or VI (purchased from EcoCyte Bioscience, Austin, TX). Then, the oocytes were kept at 18°C for 3-7 days in modified Barth's solution (NaCl 88 mM, KCl 1 mM, $NaHCO_3$ 2.4 mM, $MgSO_4$ 0.82 mM, $Ca(NO_3)_2$ 0.33 mM, $CaCl_2$ 0.41 mM, HEPES 10 mM, pH 7.4) supplemented with 10 mg/mL of gentamycin, 10 mg/mL of streptomycin. Odorant-induced currents at holding potential of -80 mV were collected from oocytes bathed in perfusion Ringer (NaCl 96 mM, KCl 2 mM, $CaCl_2$ 1.8 mM, $MgCl_2$ 1 mM, HEPES 5 mM. pH 7.6) flowing at 3.2 ml/min. For I-V curves, the holding potentials were held at -80, -60, -40, -20, 0, +20 and +40 mV. Stimulus were injected (100 µl in 2 s) at 1 cm upstream of the flow. After each stimulus, oocytes were thoroughly washed until a steady baseline was recovered. Source doses rather than the actual doses reaching oocytes are reported. Currents were amplified with an OC-725C amplifier (Warner Instruments, Hamden, CT), low-pass filtered at 50 Hz and digitized at 1 kHz. Data acquisition and analysis were carried out with Digidata 1440A and pCLAMP 10 software (Molecular Devices, LLC, Sunnyvale, CA). The panel of odorants is detailed in Table 1. N-(4-

ethylphenyl)-2-[(4-ethyl-5-pyridin-3-yl-1,2,4-triazol-3-yl)sulfanyl]acetamide (VUAA-1) was used as an Orco agonist.

REFERENCES

LEAL, W. S., BARBOSA, R. M., ZENG, F., FAIERSTEIN, G. B., TAN, K., PAIVA, M. H., GUEDES, D. R., CRESPO, M. M. & AYRES, C. F. 2017. Does Zika virus infection affect mosquito response to repellents? *Sci Rep,* 7**,** 42826.

LEAL, W. S., CHOO, Y. M., XU, P., DA SILVA, C. S. & UEIRA-VIEIRA, C. 2013. Differential expression of olfactory genes in the southern house mosquito and insights into unique odorant receptor gene isoforms. *Proc Natl Acad Sci U S A,* 110**,** 18704-9.

LIU, F., CHEN, L., APPEL, A. G. & LIU, N. 2013. Olfactory responses of the antennal trichoid sensilla to chemical repellents in the mosquito, *Culex quinquefasciatus*. *J Insect Physiol,* 59**,** 1169-77.

SYED, Z., KOPP, A., KIMBRELL, D. A. & LEAL, W. S. 2010. Bombykol receptors in the silkworm moth and the fruit fly. *Proc Natl Acad Sci U S A,* 107**,** 9436-9.

SYED, Z. & LEAL, W. S. 2008. Mosquitoes smell and avoid the insect repellent DEET. *Proc Natl Acad Sci U S A,* 105**,** 13598-603.

UEIRA-VIEIRA, C., KIMBRELL, D. A., DE CARVALHO, W. J. & LEAL, W. S. 2014. Facile functional analysis of insect odorant receptors expressed in the fruit fly: validation with receptors from taxonomically distant and closely related species. *Cell Mol Life Sci,* 71**,** 4675-80.

XU, P., CHOO, Y. M., DE LA ROSA, A. & LEAL, W. S. 2014. Mosquito odorant receptor for DEET and methyl jasmonate. *Proc Natl Acad Sci U S A,* 111**,** 16592-7.

XU, P., GARCZYNSKI, S. F., ATUNGULU, E., SYED, Z., CHOO, Y. M., VIDAL, D. M., ZITELLI, C. H. & LEAL, W. S. 2012a. Moth sex pheromone receptors and deceitful parapheromones. *PLoS One,* 7**,** e41653.

XU, P., HOOPER, A. M., PICKETT, J. A. & LEAL, W. S. 2012b. Specificity determinants of the silkworm moth sex pheromone. *PLoS One,* 7**,** e44190.

ZHU, F., XU, P., BARBOSA, R. M., CHOO, Y. M. & LEAL, W. S. 2013. RNAi-based demonstration of direct link between specific odorant receptors and mosquito oviposition behavior. *Insect Biochem Mol Biol,* 43**,** 916-23.
